# Being an older family caregiver does not impact healthcare and mortality: Data from the study ‘Good Aging in Skåne’

**DOI:** 10.1177/1403494820960648

**Published:** 2020-11-06

**Authors:** Sölve Elmståhl, Nathalie Lundholm-Auoja, Henrik Ekström, Lena Sandin Wranker

**Affiliations:** 1 Department of Clinical Sciences in Malmö, Lund University, Sweden; 2 Centre for Ageing and Health, Department of Health and Rehabilitation. University of Gothenburg, Sweden

**Keywords:** Caregiver burden, elder, epidemiology, general population, healthcare, mortality

## Abstract

**Background:**

Will being a caregiver further impact the health of a group already at risk of adverse health due to old age? This study aimed to answer the questions whether short- and long-term healthcare consumption and mortality differ between informal caregivers and non-caregivers and between high-burden and low-burden informal caregivers.

**Method:**

The study population consisted of 423 caregivers and 3444 controls from the Swedish national general population study ‘Good Aging in Skåne’. Caregivers were divided into those reporting high and low caregiver burden and information on caregiver status was collected from questionnaires. Data for mortality and healthcare consumption (inpatient and outpatient visits) were obtained from The National Board of Health and Welfare. Mortality was tested with Cox regression models and healthcare consumption with logistic regression models, adjusted for sociodemographic covariates, Activities of daily living (ADL) and number of chronic diseases.

**Results:**

Caregivers were younger than non-caregivers, had higher educational background, more independent in ADL and more often men. Of 423 caregivers, 73 (17.3%) reported experiencing high caregiver burden. High-burden caregivers were older, more dependent in personal ADL and gave more hours of care than those reporting low burden. In adjusted regression models, we found no differences in either consumption of healthcare nor mortality between caregivers and non-caregivers and high-burden v. low-burden caregivers looking at short-term (1 and 3 years) and long-term (10 and 15 years) follow-up periods.

**Conclusions:**

**Our findings suggest that the characteristic of being a family caregiver does not have an impact on mortality or physical health measured as inpatient admissions or instances of primary care.**

## Background

There are currently more than 1.3 million informal caregivers in Sweden and according to a report from the Swedish National Board of Health and Welfare, almost one out of six of those 60 years and older were identified as an informal caregiver, primarily taking care of a co-habiting spouse [1]. Informal caregiving reduces costs for home help services and assisted living facilities, but also leads to losses in form of lost tax revenue due to informal caregivers’ reduced work productivity as well as personal, financial strain put on the individual [2]. A recent review of costs of care for older adults showed that having a family caregiver reduced healthcare utilization [3].

Few studies have explored the health implications and healthcare utilization of being a caregiver and recent studies offer conflicting results where some point to caregivers being healthier, with fewer instances of inpatient care [4] as well as lower mortality rates [5, 6]. Other studies have instead shown a link between caregiving and chronic diseases such as cardiovascular disease [7] as well as increased mortality [8]. Increased mortality is mostly seen in caregivers experiencing high levels of stress or high caregiver burden, caregivers reporting low stress/burden in some studies instead being healthier than their high-stress counterparts [9, 10]. Yet other studies have not been able to find any differences in health between the two groups [11].

Recent data from the US Health Information National Trends Survey reported no difference the past year in number of healthcare appointments but called for further research on long-term morbidity [12]. In contrast, the US National Health and Welfare Survey reported higher comorbidity and number of outpatient visits the past six months (4.1 v. 2.7) among employed adult caregivers compared to non-caregivers [13]. The need for further longitudinal research is indicated in order to study, for example, the accumulation of health risk factors over time among caregivers.

This study takes a novel approach to look at health-service use and health by comparing non-caregivers with informal caregivers and high-burden informal caregivers with low-burden informal caregivers, distinguishing an important factor that may explain the lack of difference in previous research. Informal care refers to care provided by relatives or close friends in the care recipient’s home and where the caregiver is usually unpaid. Formal caregivers, who were not included in this study, usually refer to paid professional caregivers employed by the state or municipality [14].

The model of health service use by Andersen is used as a theoretical framework [15]. The model integrates predisposing, enabling and need-based factors to explain healthcare utilization. Informal caregivers are a diverse group and the varying results from studies on health and mortality could be attributed to looking at subgroups of caregivers, as it has been shown that factors such as stress [9, 16], marital status [17], gender [18, 19], educational background [20] and underlying health conditions [7, 21] all could have a possible effect on the mental and physical health of the caregiver.

The aim of the present study is to examine whether there is a difference between informal caregivers and non-caregivers and between informal caregivers with high or low burden regarding short-term (1 and 3 years) and long-term [5, 10 and 15 years) healthcare consumption and mortality during a follow-up time of 15 years in the Swedish general population aged 60 years and older.

## Methods

The study population was recruited from the prospective, longitudinal study ‘Good Aging in Skåne’ (GÅS), part of the ‘Swedish National Study on Aging and Care’ (SNAC). SNAC is a multi-centre study initiated by the Swedish Ministry of Health, studying health, illness, functional capability, life circumstances and the care need of the individual [22]. Participants of GÅS are summoned for an assessment where they undergo a medical examination, are tested regarding cognitive function and answer a comprehensive questionnaire penetrating sociodemographic data, health, activity of daily living (ADL) status, life circumstances and whether they receive or offer care, formal as well as informal. After initial assessment, participants are invited back for follow-up evaluations at regular intervals. All examinations are performed by specially trained staff comprising a physician, a registered nurse and a behavioural therapist.

Individuals included in the baseline assessment between the years 2001–2004 were randomly selected from the National Population Register from nine age cohorts: 60, 66, 72, 78, 81, 84, 87, 90 and > 93 and five municipalities representing urban and rural areas. They were invited by letter and the participation rate was 60% (*n* = 2931). Between the years 2006 and 2010 a new wave of participants aged 60 and 81 years were included, with a participation rate of 66% (*n* = 1523). All participants were evaluated adhering to the same examination protocols. From the two sets of evaluations, data from a total number of 4454 participants was collected.

**Figure 1.**
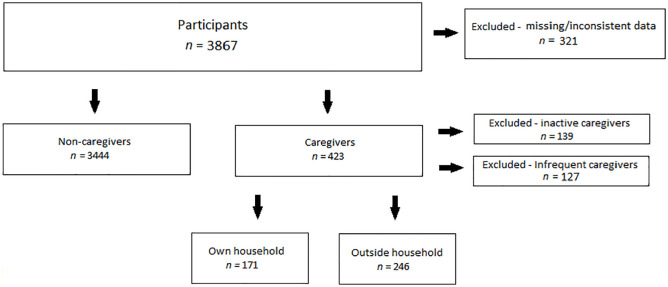
From the original study population of 4454 participants, 3867 were ultimately included in this study, of which 3444 were categorized as non-caregivers and 423 as caregivers.

At baseline, participants were categorized into ‘Caregiver’ or ‘Non-caregiver’ based on the question: ‘Do you provide care to a relative or family member?’ 3457 participants reported they were not caregivers. Of the remaining 997 participants, 308 were excluded due to data missing from the above-mentioned questions and 13 were excluded due to inconsistencies in their answers. Caregivers reporting they gave care less frequently than once per week were excluded (*n* = 127). And those reporting they had previously been a caregiver but currently were not, were also excluded (*n* = 139). This left 423 participants reporting they were currently caregivers and providing care at least once per week. Of those, 171 reported they gave care in their own home and 246 reported they gave care elsewhere, for six participants data were missing regarding where care was provided. Finally, the material consisted of 3867 participants of which 3444 were non-caregivers and 423 caregivers (see Figure 1).

Data regarding days spent in inpatient and outpatient care during inclusion in study were collected from The National Board of Health and Welfare including all counties in Sweden. Registration to the register is mandatory by law for all healthcare. Consumption of care was measured as number of days and visits admitted to inpatient care and number of outpatient visits during inclusion in the study to December 2016. Diagnosis were categorized into groups based on ICD 11. From the Mortality Register from the Swedish National Bureau of Statistics, dates of death between the 2001 and December 2016 were collected.

Informal caregiving was defined as providing unpaid care to a relative or family member who is suffering from sickness or is dependent in one or more activity of daily life. Inclusion criteria were caregivers currently providing care at least once per week. To determine caregiver burden, caregivers were asked ‘Do you feel strained by caregiving?’ with alternative answers ‘not at all”, ‘not particularly’, ‘somewhat’, ‘much’ and ‘very much’. Burden was dichotomized into high or low where high burden was defined as answering ‘somewhat’, ‘much’ and ‘very much’ to the above question [19]. Self-reported questions on affected health and health at risk were asked: ‘Do you think that your own health has been affected by the caregiving situation?’ and ‘Do you think that your own health is at risk of being affected by the caregiving situation?’ The answer options for both questions were yes/no.

The following covariates, assessed at baseline, were included to characterize caregivers/non-caregivers and used when adjusting for survival analysis: sex, age, education, number of chronic diseases, cohabiting status and ADL function. Age groups were divided into three categories: 60–69, 70–79 and > 80 years. Cohabiting status was dichotomized into ‘single’ if not co-residing with the care recipient or ‘cohabiting’ if co-residing with the care recipient. Education level was divided into three categories: elementary school or less, secondary school and one or more than one year’s university studies. Number of chronic diseases were divided into categories of 0, 1, 2 and 3 or more diseases. Cognition was assessed by the Mini-Mental State Examination scale (MMSE) and a score < 24 was categorized as cognitive decline.

Consumption of inpatient care was dichotomized as ‘high consumption’ or ‘low consumption’ with dividing point being the median number of days hospitalized between the time of entry in study and at each of the follow-up points (1, 3, 5, 10 and 15 years). The median was included in the variable ‘High consumption’. Consumption of primary care was similarly dichotomized according to the median of instances of contact with primary care.

ADL level was self-reported as well as assessed during physical examination and was rated using an ADL scale comprising nine activities: cooking, cleaning, driving/using public transportation, shopping, feeding, dressing, going to the toilet, bathing and functional mobility. ADL can further be categorized as instrumental ADL (iADL) and personal ADL (pADL) where iADL encompasses the first four items and pADL the latter five [23]. ADL status was categorized into three variables as follows: wholly independent in pADL/iADL if participants could manage all activities without any assistance; dependent in iADL if participants were independent in all pADLs but needed assistance for one or more iADL function; dependent in pADL if participants needed assistance for one or more pADL function dependent or not in iADL. Frequency of informal care and place of informal care were asked for during baseline examination.

### Statistical analysis

Chi-squared tests were used for characterization of the study population, looking at various sociodemographic and medical factors and to describe mortality and high consumption of inpatient care and primary care for the groups ‘non-caregivers’, ‘informal caregivers’ and informal caregivers reporting ‘high caregiver burden’ and ‘low caregiver burden’. Normality in age distribution in caregivers and non-caregivers and equality of variances between caregivers and non-caregivers was not examined. However, in addition to age, health status and functional capacity (ADL), as variables related to age and crucial in assessing healthcare consumption and mortality, were included in all regression models.

Cox regression models were used for survival analysis to determine whether there was a difference in mortality between caregivers and non-caregivers and between low- and high-burden caregivers. Logistic regression analysis was performed to assess any differences in consumption of inpatient and primary care between caregivers and non-caregivers and between low- and high-burden caregivers. All regression models were adjusted for sex, age, ADL status, number of chronic diseases, educational level and cohabiting status, and were performed for 3, 5, 10 and 15 years since inclusion in the study, thus looking at both shorter-term follow up (1 and 3 years) periods as well as long-term follow up (10 and 15 years) periods. A *p*-value ⩽ 0.05 was considered statistically significant. SPSS^®^ version 24 (IBM SPSS Statistics for Windows) was used for all statistical analyses.

### Ethics

Written, informed consent to participate in the study, to access register data and healthcare journals were obtained from all participants. This study was conducted in accordance with the World Medical Association’s (WMA) Declaration of Helsinki (developed as a statement of ethical principles for medical research). The GÅS has been approved by the Ethics Board at Lund University, Lund (LU 744-00).

## Results

In the sample of 3867 participants 44.8% were men and 55.2% female. We found 10.9% (*n* = 423) identified as caregivers and of those of 19.1% (*n* = 73) reported high burden, 19.2% (*n* = 14) reported ‘very much strain’ and 80.8% (*n* = 59) ‘much strain’. In the low-burden group, 80.9% (*n* = 310) of the caregivers, 48.4% (*n* = 150) reported ‘not at all strained’, 32.3% (*n* = 100) ‘not particularly strained’ and 19.45% (*n* = 60) ‘somewhat strained’.

A higher percentage of men than women identified as caregivers (52.5 and 47.5% respectively). Caregivers were slightly younger than non-caregivers with a mean age of 68.3 years compared to a mean age of 70.4 (*p* < 0.001) and they were more independent in ADL than non-caregivers (*p* = 0.001). Caregivers reporting higher burden were older and less independent in ADL than those reporting low burden (*p* = 0.047 and *p* = 0.008, respectively) (Table I).

**Table I.** Sociodemographic and health characteristics at baseline for caregiver and non-caregivers and in caregivers reporting high and low burden from the Swedish general population study ‘Good Aging in Skåne’ (GÅS).

Variables at baseline	Non-Caregivers *n* = 3444	Caregivers *n* = 423	*p*	Low burden *n =* 310	High burden *n* = 73	*p*
Sex, ***n*** (%)						
Male	1512 (43.9)	222 (52.5)	0.001	173 (55.8)	32 (43.8)	0.065
Female	1932 (56.1)	201 (47.5)		137 (44.2)	41 (56.2)	
Age, ***n*** (%)						
60–69 years	1995 (57.9)	285 (67.4)	0.001	216 (69.7)	40 (54.8)	0.047
70–79 years	451(13.1)	44 (10.4)		32 (10.3)	10 (13.7)	
80+ years	999 (29.0)	94 (22.2)		62 (20.0)	23 (31.5)	
Mean, years (SD)	70.4 (10.4)	68.3 (9.5)	< 0.001	67.8 (9.3)	70.3 (10.0)	0.042
Education, ***n*** (%)						
Elementary	1651 (47.9)	151 (35.7)	< 0.001	117 (37.7)	29 (39.7)	0.054
Secondary	955 (27.7)	129 (30.5)		106 (34.2)	17 (23.3)	
University	739 (21.5)	114 (27.0)		87 (28.1)	26 (35.6)	
Missing	99 (2.9)	29 (6.8)			1 (1.4)	
Cohabiting Status, ***n*** (%)						
Cohabiting	2067 (60.1)	327 (77.3)	< 0.001	236 (76.1)	14 (19.2)	0.391
Single	1373 (39.9)	96 (22.7)		74 (23.9)	59 (80.8)	
ADL, ***n*** (%)						
Independent	1923 (55.8)	265 (62.7)	0.001	219 (70.6)	40 (54.8)	0.008
iADL depend	432 (12.5)	38 (9)		30 (9.7)	4 (5.5)	
pADL depend	880 (25.6)	81 (19,1)		56 (18.1)	24 (32.9)	
Missing	209 (6.1)	39 (9.2)		5 (1.6)	5 (6.8)	
Chronic disease, ***n*** (%)						
0	1087 (31.6)	132 (31.2)	0.016	105 (33.9)	22 (30.1)	0.682
1	1081 (31.4)	128 (30.3)		102 (32.9)	22 (30.1)	
2	634 (18.4)	93 (21.9)		72 (23.3)	19 (26.0)	
3 or more	430 (12.5)	32 (7.6)		23 (7.4)	8 (11.0)	
Missing	212 (6.1)	38 (9.0)		8 (2.6)	2 (2.8)	
MMSE < 24, ***n*** (%)	359 (11.0)	26 (6.7)	0.010	61 (19.7)	9 (12.3)	0.031
Informal care, ***n*** (%)						
1–3 times/week		222 (52.5)	< 0.001	184 (59.4)	24 (32.9)	< 0.001
4–7 times/week		164 (38.8)		112(36.1)	46 (63.0)	
Missing		37 (8.7)		14(4.5)	3 (4.1)	
Place of care, ***n*** (%)						
Own home		171 (40.4)		117 (37.7)	40 (54.8)	0.037
Outside home		246 (58.2)		178 (57.4)	32 (43.8)	
Missing		6 (1.4)		15 (4.9)	1 (.4)	
Health affected? ***n*** (%)						
Yes		36 (8.5)	0.460	9 (2.9)	26 (35.6)	< 0.001
No		329 (77.8)		283 (91.3)	46 (63.0)	
Missing		58 (13.7)		18 (5.8)	1 (1.4)	
Health at risk? ***n*** (%)						
Yes		59 (14.0)	0.380	23 (7.4)	35 (47.9)	< 0.001
No		305 (72.0)		269 (86.8)	35 (47.9)	
Missing		59 (14.0)		18 (5.8)	3 (4.1)	

ADL: activity of daily living; iADL: instrumental ADL; pADL: personal ADL; MMSE: Mini-Mental State Examination scale.

The amount of time invested in caregiving played a role in reported burden, where 63% of the group offering care four or more times/week reported high burden compared to 36% in the low burden group (*p* < 0.001) (Table I).

At the end of follow-up time (15 years), 38.0% of all participants, caregivers and non-caregivers were deceased. Looking at follow-up times of 5, 10 and 15 years, non-caregivers had higher mortality compared to caregivers. At all follow-up times, a slightly higher percentage of high-burden caregivers had died compared to low-burden caregivers, this was however not statistically significant (Table II).

**Table II.** Numbers and proportions of mortality and high consumption of inpatient care and primary care comparing non-caregivers and caregivers as well as caregivers reporting high or low burden from the Swedish general population study ‘Good Aging in Skåne’ (GÅS).

Outcomes	Non-Caregivers *n* = 3444	Caregivers *n* = 423	*p*	Low burden *n =* 310	High burden *n* = 73	*p*
Mortality 5 years, ***n*** (%)	514 (14.9)	37 (8.7)	0.001	26 (8.4)	8 (11.0)	0.487
Mortality 10 years, ***n*** (%)	1039 (30.2)	102 (24.1)	0.010	75 (24.2)	19 (26.0)	0.743
Mortality 15 years, ***n*** (%)	1333 (38.7)	138 (32.6)	0.015	103 (33.2)	25 (34.2)	0.868
Inpatient care^a^, ***n*** (%)						
1 year	510 (14.8)	54 (12.8)	0.261	38 (12.3)	9 (12.3)	0.987
3 years	1221 (35.5)	123 (29.1)	0.009	93 (30.0)	19 (26.0)	0.502
5 years	1664 (48.3)	167 (39.5)	0.001	130 (41.9)	25 (34.2)	0.229
10 years	1765 (51.2)	195 (46.1)	0.046	151 (48.7)	23 (43.8)	0.453
15 years	1751 (50.8)	189 (44.7)	0.017	146 (47.1)	30 (41.1)	0.355
Primary care^b^, ***n*** (%)						
1 year	1569 (45.6)	182 (43.0)	0.324	135 (43.5)	35 (47.9)	0.496
3 years	1873 (54.5)	224 (53.0)	0.578	161 (51.9)	46 (63.0)	0.088
5 years	1803 (52.4)	218 (51.5)	0.751	160 (51.6)	39 (53.4)	0.780
10 years	1788 (51.9)	230 (54.4)	0.340	174 (56.1)	42 (57.5)	0.828
15 years	1792 (52.0)	227 (53.7)	0.526	176 (56.8)	40 (54.8)	0.759

a High consumption defined as above median days of inpatient care at follow-up time.

b High consumption defined as above median number of visits at primary care at follow-up time.

High consumption of inpatient care, in unadjusted, logistic regression models, was less frequent in caregivers compared to non-caregiver and most pronounced after 3 and 5-year follow-up time (Table II). The percentage of high-consumption among caregivers was 29.1% at 3 years follow-up time and 39.5% at 5 years follow-up time compared to respectively 35.5 and 48.3% for non-caregivers. The median of number of inpatient admissions for non-caregivers and caregivers were the same for the follow-up periods 1, 3, 5 and 15 years, median values 0, 2, 4 and 10 inpatient admissions, respectively. Median inpatient admissions for year 10 follow up were 9 for caregivers and 8 for non-caregivers.

Primary-care consumption did not differ between caregivers and non-caregivers. Median numbers of visits at primary care were the same for caregivers and non-caregivers for all 1, 3, 5, 10 and 15-year follow-up periods, with respectively 0, 2, 4, 8 and 10 median visits.

In comparison to low-burden caregivers, those caregivers reporting high burden had numerically fewer days of hospital admission at 3, 5, 10 and 15 years, but more instances of primary care at 1, 3, 5 and 10-year follow-up time, but these numbers where not statistically significant (Table II).

In Cox regression models adjusted for age, sex, education, cohabiting status, ADL function and number of chronic diseases, there was no significant difference in mortality between caregivers and non-caregivers at all follow-up periods from 1 to 15 years (Table III). In adjusted logistic regression models, there was no difference between caregivers and non-caregivers regarding inpatient-care or primary-care consumption (Table IV).

**Table III.** Cox regression models analysing mortality in caregivers compared to non-caregivers as well as in caregivers reporting high or low burden. Models are adjusted for sex, age, education level, cohabiting status, activity of daily living (ADL) and number of chronic diseases, looking at follow up times of 1, 3, 5, 10 and 15 years.

Mortality	Caregivers^a^			*p*	High-burden caregivers^b^			*p*
	B coeff.	HR	95% CI		B coeff.	HR	95% CI	
1 year	0.081	1.084	0.424–2.772	0.866	−0.842	0.431	0.046–4.051	0.462
3 years	−0.124	0.883	0.533–1.463	0.630	−0.593	0.553	0.184–1.660	0.291
5 years	−0.116	0.891	0.623–1.274	0.527	−0.040	0.961	0.420–2.198	0.924
10 years	0.102	1.108	0.890–1.378	0.359	0.293	1.340	0.780–2.304	0.289
15 years	−0.022	0.979	0.812–1.180	0.821	0.325	1.383	0.861–2.224	0.180

CI: confidence interval.

a Non-caregivers as reference.

b Low-burden caregivers as reference.

**Table IV.** Multivariate logistic regression analysis looking at consumption of inpatient care and primary care for caregivers compared to non-caregivers as well as for caregivers reporting high or low burden. Models are adjusted for sex, age, education level, cohabiting status, activity of daily living (ADL) and number of chronic diseases.

	Consumption inpatient care			*p*	Consumption primary care			*p*
	B coeff.	OR	95% CI		B coeff.	OR	95% CI	
1 year								
Caregivers^a^	0.029	1.030	0.735–1.443	0.866	0.067	1.069	0.856–1.337	0.555
High burden^b^	0.466	1.594	0.615–4.134	0.338	0.016	1.016	0.582–1.772	0.956
3 years								
Caregivers^a^	0.076	1.079	0.841–1.386	0.549	0.076	1.079	0.866–1.346	0.497
High burden^b^	0.541	1.718	0.861–3.429	0.125	−0.359	0.698	0.395–1.234	0.217
5 years								
Caregivers^a^	0.142	1.152	0.911–1.458	0.238	0.053	1.054	0.846–1.313	0.639
High burden^b^	0.639	1.894	1.017–3.525	0.044	0.027	1.028	0.588–1.795	0.923
10 years								
Caregivers^a^	−0.070	0.932	0.735–1.182	0.563	−0.092	0.912	0.730–1.139	0.415
High burden^b^	0.456	1.577	0.843–2.950	0.154	0.012	1.012	0.574–1.783	0.968
15 years								
Caregivers^a^	−0.024	0.976	0.768–1.240	0.842	−0.069	0.934	0.747–1.166	0.545
High burden^b^	0.402	1.495	0.807–2.771	0.202	0.078	1.081	0.613-1.907	0.787

CI: confidence interval; OR: odds ratio.

a Non-caregivers as reference.

b Low-burden caregivers as reference.

## Discussion

The results from this general population study did not demonstrate that being a family caregiver have any short-term or long-term adverse impact on mortality or healthcare consumption. Caregivers with high burden had no different mortality or primary care use at short- or long-term follow-up, but higher inpatient care at 5-year follow-up.

In this material, 10.9% participants were identified as a caregiver compared to the Swedish national average of approximately 14% for the same age cohorts and the same degree of informal support once a week or more [1, 24].

Worldwide, women outweigh men when it comes to caregiving [25], but in Sweden a recent national survey indicated that men and women provide informal care to the same extent [1]. In this study male caregivers outweighed female caregivers. A possible explanation could be attributed to under-reporting of caregiver status, traditional gender roles resulting in more female caregivers looking at the provided care as a natural part of their duties and therefore not identifying themselves as a caregiver [26].

In line with previous findings, caregivers reporting high burden gave more care and more often gave care in their own home, as well as being older and reporting dependence in pADL to a higher extent than low-burden caregivers. Co-residing caregivers are likely to provide more hours of care than those giving care outside their own home [27] and more often report feelings of confinement and experiencing limitations in everyday life [28]. It is therefore natural for them to experience caregiving as more of a burden than those who do not live with the care recipient.

Compared with non-caregivers, caregivers were younger (2 years) and a larger proportion (62.7 v. 55.8%) were independent of ADL. Among caregivers, the proportion of independent of ADL decreased on average about 0.8% per year. The difference in age between caregivers and non-caregivers would thus only partly explain a larger proportion of ADL independence among caregivers. The difference in ADL dependence between caregivers and non-caregivers, could rather be explained by the workload it entails to be a caregiver.

The 2012 report from The National Board of Health and Welfare found that, in the Swedish population, those from lower educational backgrounds more often were caregivers and provided more extensive care than individuals with higher education [1]. It is interesting that our results show the inverse relationship: caregivers were shown to have a higher educational background than non-caregivers (Table I). Even if the participants were randomized from the population register, we cannot rule out that a selection bias may be the reason why a larger proportion of highly educated participants were found among caregivers. However, a more possible explanation is that a larger proportion of caregivers were men (Table I), and men were in general better educated. Among caregivers with secondary school or university studies 57.2% were men and 42.8% were women.

Caregiving status of a participant is likely to change during a study, either due to the care recipient dying, the care level fluctuating or due to a previous non-caregiver transitioning into caregiver. In unpublished material from GÅS, 51.3% of baseline caregivers had transitioned out of caregiver status during a six-year follow up, whereas 10.9% of non-caregivers had become caregivers during the same time period. So, if caregiver status can change, potential accumulation of health-risk effects of being a caregiver could theoretically be evened out due to fluctuating caregiver status during a study with a long follow-up time. We have determined caregiver status at baseline assessment, and to account for this, we have looked at both shorter term and longer term follow-up periods of 1, 3, 5, 10 and 15 years.

Many studies that yield results showing differences between caregivers’ and non-caregivers’ health and mortality also tend to only look at subgroups of caregivers: studying female caregivers, those providing care to relatives with specific diagnoses like dementia or stroke or caregivers themselves exhibiting various symptoms or diagnoses. This limits the possibilities to generalize data to the general population from a healthcare planning perspective. To our knowledge, only a few papers have studied actual healthcare consumption as a measure of caregiver health, studies more often looking at self-reported health or stress [18, 19].

A previous model of health-service use was developed by Andersen to understand the use of formal health service, including predisposing, enabling and need-based factors [15]. The predisposing factors included age, gender and education; enabling factors included cohabiting status, place of care, health-service organization and social regulation; and need factors included own view of general health, functional status and chronic diseases. In this study these factors have been included in the adjusted model to explain variation in healthcare utilization and mortality. We found no negative impact of being a caregiver regarding neither mortality nor healthcare consumption and the results are in line with a study from the USA looking at co-residing caregivers that showed caregivers had worse self-reported health compared to their controls, but found caregivers had slightly lower inpatient admissions than non-caregivers [4]. Our results are also in line with another, recent study on caregivers in Gunma, Japan, that found no difference in hospital admission between caregivers and non-caregivers [11].

Our findings do not negate any deleterious health effects that caregiving may have on certain groups of informal caregivers. Instead, they highlight the need to better identify which categories of caregivers are indeed at risk, allowing for more accurately directed preventive measures and intervention strategies. Venues for further studies could be looking at whether place of caregiving (at home/outside own home), or the relationship to the care recipient (parent, child, spouse or other) has an impact on mortality or morbidity for the caregiver. High-burden caregivers had higher consumption of inpatient care at 5-year follow up, and though not statistically significant, our numbers suggest high-burden caregivers have more contact with primary care but fewer days of inpatient care than low-burden caregivers, providing material for future study. We have previously reported that the specific diagnosis of the subject receiving support from the next of kin have impact on the caregiver burden, especially depression and dementia [21]. The primary independent variable in this study was being a caregiver, irrespectively of other support like having a formal caregiver financed by the municipality. A previous published study using the same data set has shown that formal caregiving varies between different diagnosis of the care recipient from 77% in fracture diagnosis to 23% among recipients with a depression, and no association was noted between those receiving a high degree of support for pADL and iADL such as stroke, heart and lung disease and fracture and the degree of high caregiver burden [21].However, the highest proportion of high caregiver burden was noted among informal caregivers helping care recipients with dementia.

One strength of this study is that the participants were randomized from a general and large population-based sample linked with longitudinal register data on health consumption and mortality and that the participation rates were high: 60 and 66% randomization respectively. Another strength is the completeness of healthcare consumption, since registration of inpatient and outpatient visits in Sweden is mandatory by law irrespective if it is a public or private healthcare provider.

Nevertheless, a participation rate of 60 to 66% opens up questions of possible selection bias. Home visits were offered to include those too frail, or otherwise uninclined to partake in the study, but there is still a possible risk of excluding participants, for example those giving extensive care since they have neither the time nor energy to participate, possibly explaining the somewhat lower number of caregivers in this study

## Conclusions

More men than women reported being a caregiver and caregivers were overall younger and more independent in ADL than non-caregivers. Caregivers reporting high burden were more likely to provide more care, care in their own home, were more dependent on ADL and slightly older. In adjusted regression we found neither differences between caregivers and non-caregivers nor high-burden and low-burden caregivers regarding consumption of inpatient and outpatient healthcare and mortality looking at the short- (1 and 3 years) and long-term (10 and 15 years) follow up after inclusion in the study. These findings suggest that the characteristic of being a caregiver does not have an adverse impact on mortality and physical health.
